# Research on a Novel Low Modulus OFBG Strain Sensor for Pavement Monitoring

**DOI:** 10.3390/s120810001

**Published:** 2012-07-25

**Authors:** Chuan Wang, Qingli Hu, Qiyu Lu

**Affiliations:** School of Civil Engineering, Harbin Institute of Technology, Harbin 150090, China; E-Mails: huqingli@hit.edu.cn (Q.H.); lu_qi_yu@163.com (Q.L.)

**Keywords:** OFBG, strain measuring, low modulus, asphalt concrete

## Abstract

Because of the fatigue and deflection damage of asphalt pavement, it is very important for researchers to monitor the strain response of asphalt layers in service under vehicle loads, so in this paper a novel polypropylene based OFBG (Optical Fiber Bragg Gratings) strain sensor with low modulus and large strain sensing scale was designed and fabricated. PP with MA-G-PP is used to package OFBG. The fabrication techniques, the physical properties and the sensing properties were tested. The experimental results show that this kind of new OFBG strain sensor is a wonderful sensor with low modulus (about 1 GPa) and good sensitivity, which would meet the needs for monitoring some low modulus materials or structures.

## 1. Introduction

Fatigue is one of criteria in asphalt concrete pavement design, and the fatigue failure of asphalt concrete has a correlation with the magnitude of tensile strain at the bottom of the asphalt layer [1–3]. Therefore, it is very important to monitor the strain response of asphalt layers in service under vehicle loads [4,5]. However, the existing methods are very limited in measuring the strain changes for the low modulus of asphalt concrete, which is approximately 1 GPa. Traditional electronic strain guages have difficulties to work when the insulation and electromagnetism are concerned. Optical Fiber Bragg Gratings (OFBG) sensors have been widely used in civil infrastructures in recent years [6–9], so some researchers have turned to use optical fiber sensors due to their vibration resistance and electromagnetism immunity [10–12]. However, the modulus of the traditional optical fiber sensor is so high that it is very hard to obtain the real strain changes of the asphalt pavement [13–15].

In this research, a novel polypropylene based OFBG strain sensor with low modulus and large strain sensing scale was designed and fabricated. Because it has almost the same modulus and constitutive relationship of strain and stress with asphalt, PP (polypropylene) can be regarded as a proper matrix in packaging bare OFBG to fabricate strain sensors to monitor the strain response of asphalt concrete pavement. In this study, the physical and mechanical performance of the PP resin matrix was studied; the appropriate fabrication techniques were developed and the sensing properties of this kind of sensor were calibrated.

## 2. Materials

### 2.1. Material Properties

PP with different contents of maleic anhydride graft PP was selected as the matrix materials for the OFBG in this research. The properties of PP are shown in Table 1, and the properties of MA-G-PP (maleic anhydride graft polypropylene) are shown in Table 2. The sensing coefficients of strain and temperature of the OFBG are 1.2 pm/με and 10 pm/°C, respectively.

### 2.2. Rheology Performance of the Resin Matrix

**Figure 1.**
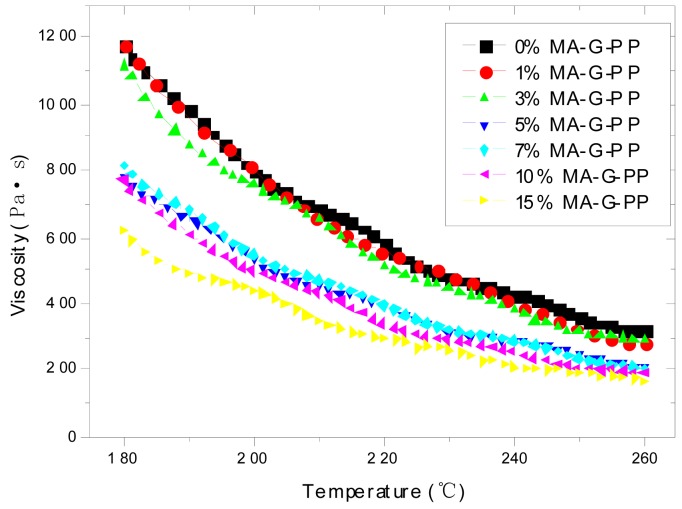
Variation of apparent viscosity of PP. matrix (0%∼15%) with temperature.

The rheology behaviors of seven kinds of PP with different contents of MA-G-PP were tested using the AR2000 rheometer produced by TA Instruments. It can be seen from Figure 1 that the apparent viscosity of melting PP is very sensitive to temperature. With the temperature raises from 180 °C to 260 °C, the viscosity decreases to about the 1/3 of the original value of each specimen. The higher the content of the MA-G-PP, the lower the viscosity is. For low levels of MA-G-PP content, such as 0%, 1% and 3%, the change of viscosity is not apparent. A sudden drop appears for the 5% content, while 7%, 10% and 15% show almost identical results as the former. The viscosity of the PP with 5% of MA-G-PP is much lower than that of the PP without MA-G-PP, as shown in Figure 1. Concerning both the viscosity and the degradation of PP, 220 °C is a proper operating temperature.

**Figure 2.**
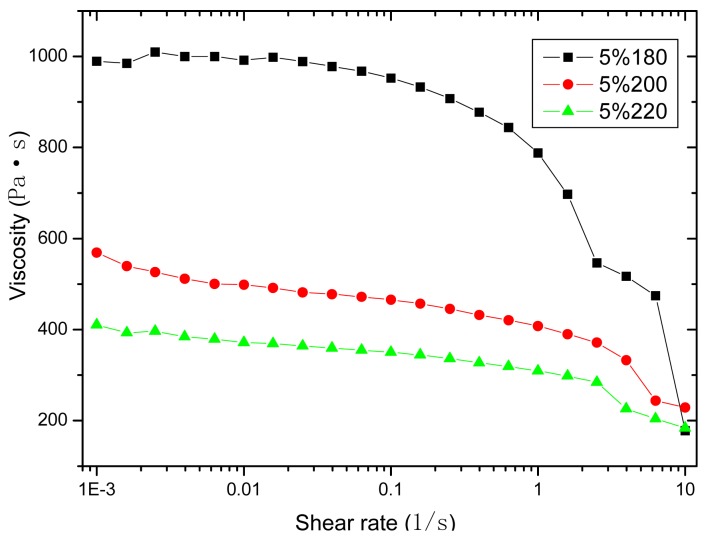
Variation of apparent viscosity of PP. matrix (5%) with shear rate.

It can be seen in Figure 2 that melted PP with different contents of MA-G-PP is a typical non-Newtonian fluid, and the viscosity decreases as the shear rate ascends. Concerning both the rheology behavior and the mechanical performance, the PP with 5% content of MA-G-PP was chosen as the proper matrix in this research to package the bare OFBG.

## 3. Fabrication of the PP Based OFBG Strain Sensor (Processing Techniques)

### 3.1. Interface Strain Transfer of PP and OFBG

The idea of the paralleled semi-extension rule based algorithm is as follows: firstly, the algorithm decomposes the maximum terms space of the clause set into several partial maximum terms spaces, which convert the SAT problem of the clause set into the SAT problem of the partial maximum terms spaces. If there is a certain partial maximum terms space that is satisfied, then the clause set is satisfied. If all the partial maximum terms spaces are unsatisfied, then the clause set is unsatisfied. In other words, the clause set is satisfied. In the following, the concept of the partial maximum terms space is discussed.

The internal force of PP and OFBG can be calculated as follows:

(1) $$\sigma_{p}=\frac{\varepsilon_{sp}}{\frac{1+C}{E_{p}}+\frac{A_{p}}{A_{f}E_{f}}}$$

where: *σ_p_, E_P_* and *A_p_* represent the internal stress, modulus and sectional area of PP matrix, respectively; *ε_sp_* is the free shrinkage strain of PP matrix without any restraint. *E_f_* and *A_f_* refer to the modulus and sectional area of optical fiber, respectively, *C* is creep coefficient.

Considering the size of sensor designed in this study, *σ_p_* should be very small, which is about 0.0832 Pa. The creep of the sensor should not be significant and the strain in the sensor can remain the same level as that in the concrete or asphalt pavement. Therefore, it can test the real strain changes.

**Figure 3.**
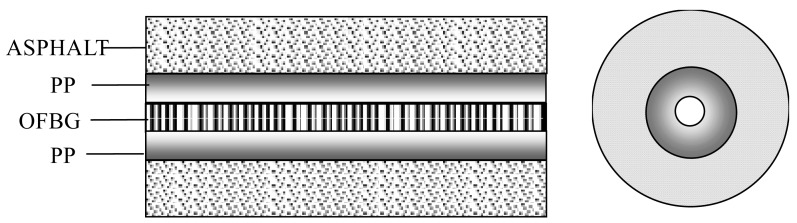
Bare FBG directly embedded in structure.

According to the model given by Ou and Zhou [16], the strain transfer performance of the interface between the fiber and PP as shown in Figure 3 can also be expressed by the following equation:

(2) $$\lambda^{2}=\frac{2G_{\text{PP}}}{E_{\text{c}}{r_{\text{c}}}^{2}\ln\frac{r_{\text{PP}}}{r_{\text{c}}}}$$

where *λ*—strain transfer eigenvalue, *E*_c_—modulus of OFBG, *r*_c_—radius of OFBG, *G*_pp_—shear modulus of the matrix, *r*_pp_—radius of the matrix.

So, the error rate of the strain transfer of the interface between OFBG and matrix (*η*) and correction factor (k) can be described as:

(3) $$\eta =\frac{\text{ch}(\lambda l_{\text{f}})-1}{\lambda l_{\text{f}}\;\text{sh}(\lambda l_{\text{f}})}$$

(4) $$k=\frac{1}{1-\eta }$$

**Figure 4.**
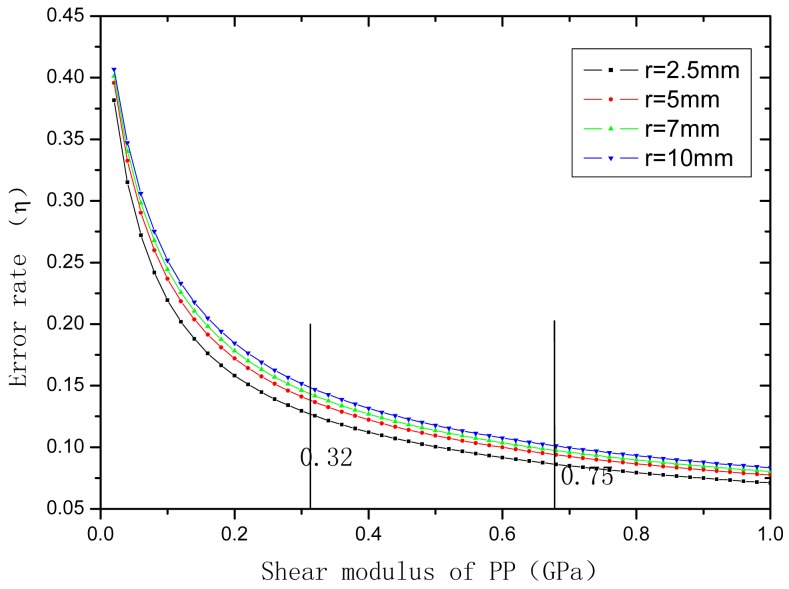
Effect of shear module on strain transfer error coefficient.

**Figure 5.**
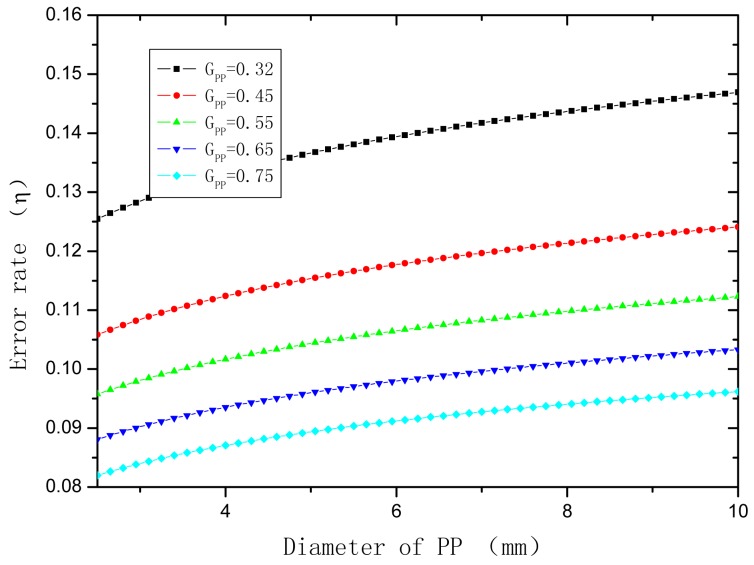
Effect of semi-radius on strain transfer error coefficient.

The shear modulus of PP ranges from 0.32 to 0.75 GPa, and the diameter of sensor is 5∼20 mm. *E*_c_ is about 70 GPa and *r*_c_ is about 6.25 × 10^−2^ mm, the l_f_ is 10 mm. The relationship of *η* and *G*_pp_ and *r*_pp_ is shown in Figures 4 and 5, respectively.

With the *G*_pp_ (0.4 Gpa) and *r*_c_ (14 mm), the error rates of the strain transfer of the interface between OFBG and matrix (*η*) and correction factor (k) are 0.126898 and 1.145342, respectively, and the strain sensing coefficient is 1.04 pm/με.

### 3.2. Fabricating Techniques

The rate of extrusion for the PP based OFBG strain sensor is set as 16 r/min. The temperature settings of the extruder are shown in Table 3.

**Figure 6.**
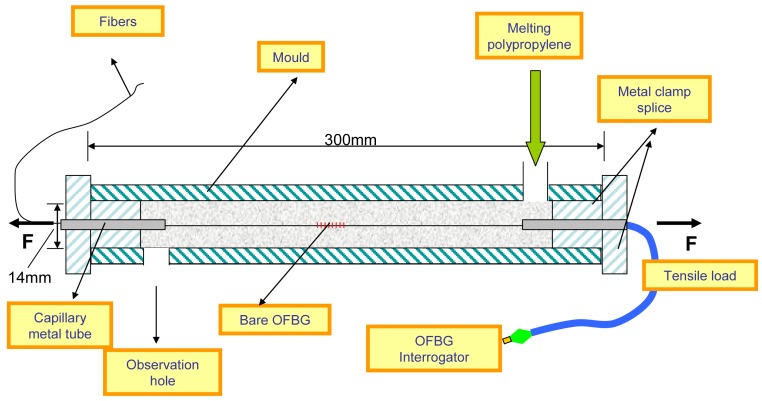
Schematic of the fabricating process of PP-OFBG strain sensor.

**Figure 7.**
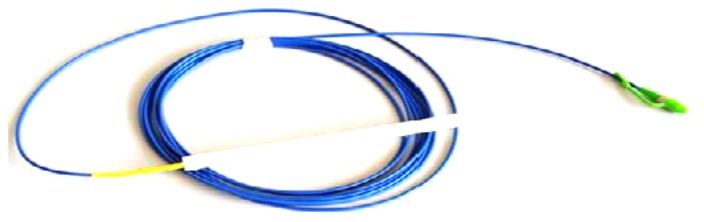
PP packaged OFBG strain sensor.

The fabrication process includes the following four steps as shown in Figure 6: (1) PP matrix is melted and extruded from the extruder; (2) Melted PP matrix enters a mould with a bare OFBG fixed along the axis; (3) The mould is put into a cooling water channel to harden the PP matrix; (4) Open the mould and the sensor is obtained. The PP-packaged OFBG strain sensor is as shown in Figure 7.

### 3.3. Hardening Process Monitoring of PP Based OFBG Strain Sensor

OFBG have been used for monitoring FRP or concrete inner strain changes during their hardening process, and valuable conclusion were obtained [17,18]. Thus, during the fabricating of the sensor, the inner strain and temperature changes of the PP matrix with the OFBG were monitored to ensure that the bare OFBG was bonding well with the PP matrix.

**Figure 8.**
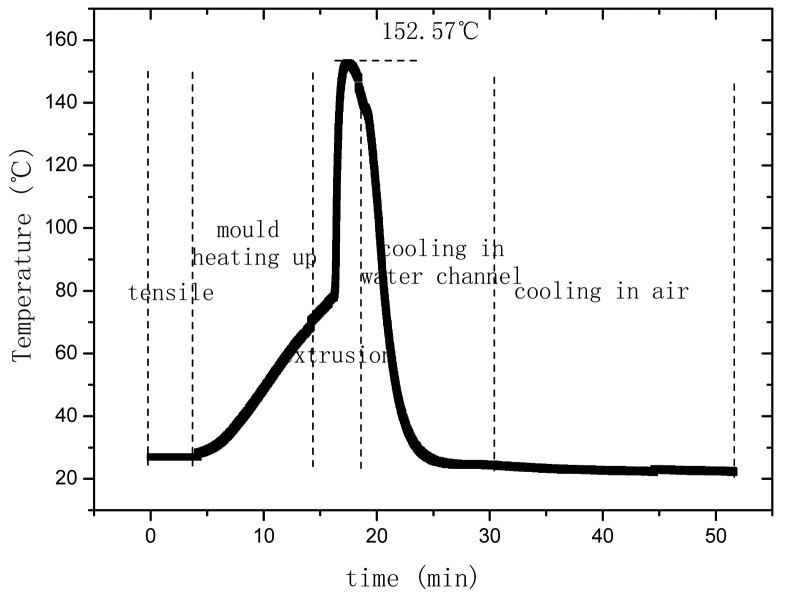
Temperature-time relationship of OFBG.

**Figure 9.**
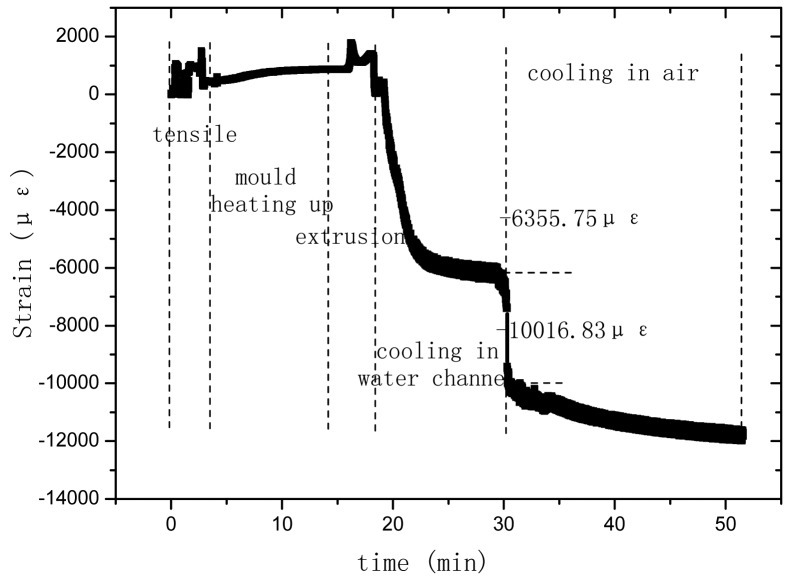
Strain-time relationship of OFBG.

The temperature-time relationship of OFBG is shown in Figure 8. The extrusion temperature of PP was originally set at about 220 °C. After PP went inside the mould and reached the OFBG position, the temperature of PP became 152.57 °C and continued descending. This would increase the viscosity of PP and hence, the viscous force increases accordingly. Figure 9 shows the strain changes of the OFBG, and from which it can be seen that the shrinkage of PP is very large. By hardening for 50 minutes, the inner strain reached −12,000 με. Three days later, the wavelength was stable at about 1,524,320 nm, and the inner strain change was about −13,060 με, which truly reflected the shrinkage of PP. Because of the high ultimate strain of PP, this sensor maybe suitable to monitor large scale tensile strains.

## 4. Tensile Sensing Test of the PP Based OFBG Strain Sensor

**Figure 10.**
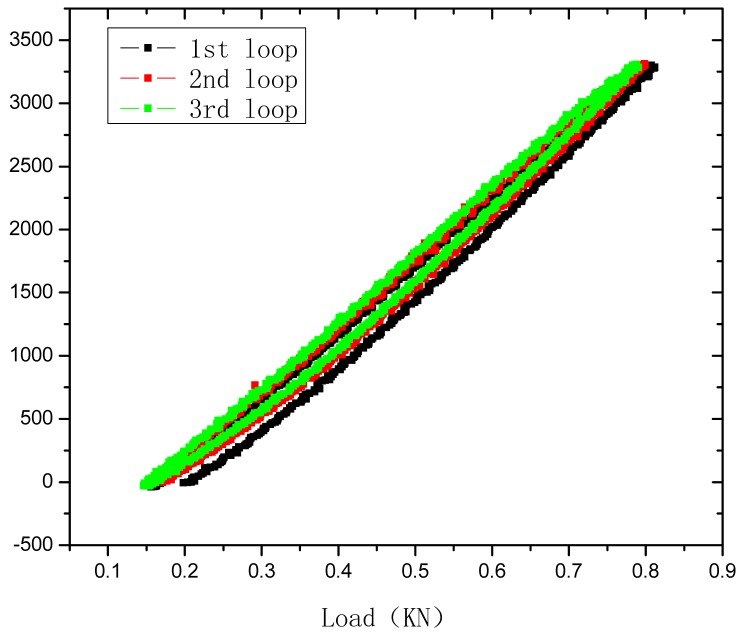
Strain-load relationship of PP-OFBG sensor.

**Figure 11.**
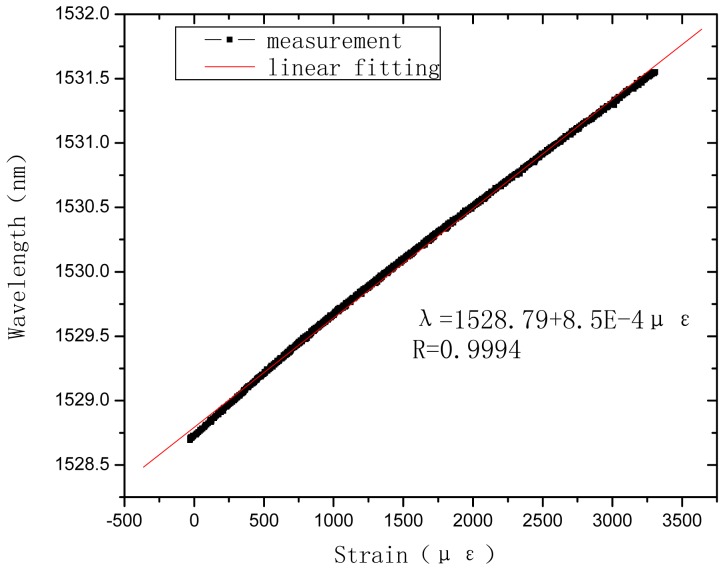
Strain sensing properties of PP-OFBG sensor.

**Figure 12.**
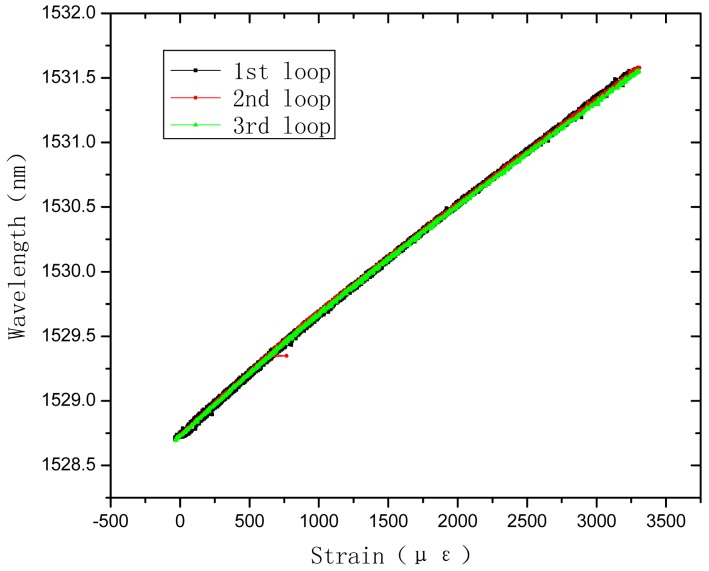
Repetitive properties of PP-OFBG sensor strain sensing.

The tensile sensing tests were carried out using an universal testing machine. The load measurement precision is 0.001 kg. A 50 mm extensometer was used to measure the tensile strain of the sensor. The strain measurement precision is 0.001 mm. The calibration test is shown as Figure 10. The wavelength of OFBG was measured with the FBG-SLI Interrogator SI720 of Micron Optics. The load, tensile strain, and wavelength of OFBG were recorded, as shown in Figures 11 and 12. Because the PP matrix is in an elastomeric state with a low modulus like asphalt at normal atmospheric a temperature, time-lag phenomenon appears, but the repetitive properties are very good, so it can be used in monitoring low modulus materials or structures.

It can be seen in Figure 11 that the sensing coefficient of this new sensor is about 0.85 pm/με. The calculation result in Section 3.1. is about 1.04 pm/με. The sensibility factor of OFBG is 1.20 pm/με. The difference among them is due to the difference between the micro-mechanics interface strain transfer performance and the materials' actual performance. To verify the repetitive properties of this new kind of sensor, the specimens were subjected to several loading and unloading cycles. The test results reveal that PP-OFBG strain sensor can work very well in load-unload loops because it is in the elastic range, as shown in Figure 12.

## 5. Concrete and Asphalt Concrete Test

### 5.1. Compressive Test in Concrete Column

**Figure 13.**
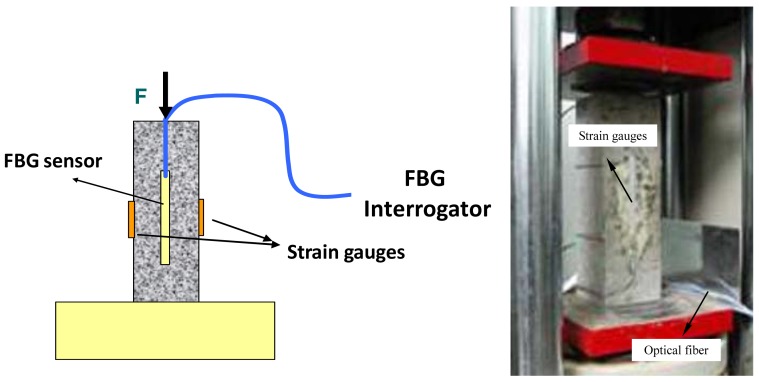
Compressive test of concrete column.

**Figure 14.**
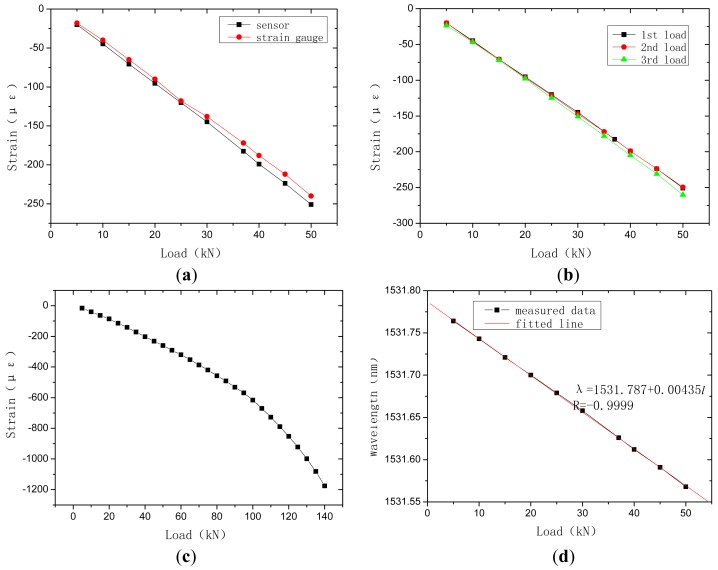
Compressive test results of concrete column: (**a**) Comparison of the developed sensor and resistance gauge; (**b**) Load-strain curve of three load-unload cycle; (**c**) Load-strain curve to failure; (**d**) Wavelength-load curve of the developed sensor.

Axial compressive test is performed to concrete column embedded with the proposed sensor to monitor strain under different load. In order to comparison, resistance strain gauges are also stuck on the lateral sides of the concrete column as shown in Figure 13. The test using load instrumentation were conducted obtain force of three load-unload cycle in elastic stage and the course to failure. The results are shown in Figure 14.

Figure 14(a) shows the strain curves of the developed sensor and resistance gauge. It can be seen that they are similar. Due to the high stiffness of resistance gauges, strain measured by them is a bit smaller. Three curves under three load-unload cycles in Figure 14(b) possess good repeatability and linearity. It can be verified that stickiness between optical fiber Bragg grating and polypropylene, sensor and concrete are all reliable. The whole response in Figure 14(c) correlatively tracks the course of load and strain. When the strain is less than 600 με, elastic deformation arises. When the strain reaches 1,200 με, failure occurs. Correlative curve between load and central wavelength of the developed sensor is shown in Figure 14(d). It is almost a straight line with a proportion coefficient of 4.35 pm/kN and correlative coefficient of 0.9999.

### 5.2. Test in Asphalt Concrete Beam

**Figure 15.**
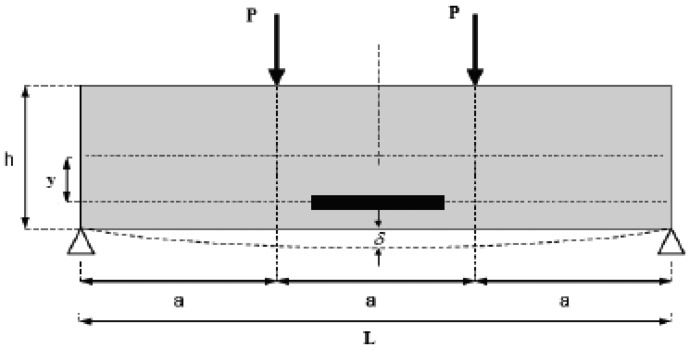
Test of asphalt concrete beam.

**Figure 16.**
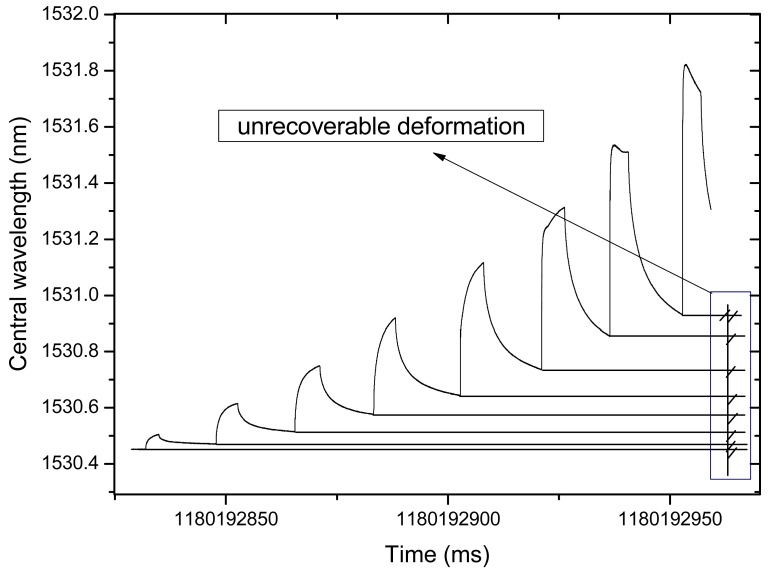
Static load experiment monitoring results of PP-OFBG strain sensor.

Steel dies with inner size of 100 mm × 100 mm × 400 mm were prepared. The new developed sensor is embedded after 10 mm thick asphalt concrete is spread. The following operation is the same as the fabrication of regular asphalt specimens. The MTS810 test machine is used to press the beam as shown in Figure 15. The load is from 0.5 KN∼5 KN, and the steps are 0.5 KN. Two displacement transducers are also installed to measure the flexibility of the beam. According to the monitoring results, we can see that the deformation of the asphalt specimen can be divided into two parts, recoverable and unrecoverable, as shown in Figure 16.

The strain changes of the asphalt specimen can be calculated according to the knowledge of materials mechanics:

$$\varepsilon_{\text{sensor}}=\varepsilon \times \frac{y}{\left(\frac{h}{2}\right)}=\frac{108\delta h}{23L^{2}}\times \frac{2y}{h}=\frac{24\delta }{23a^{2}}y$$

where: *a* = *h* = *(1/3)L, y* = *(2/5)h*=*(2/15)L*. And δ can be obtained by two methods. One is by MTS, and the other is by LVDT.

**Figure 17.**
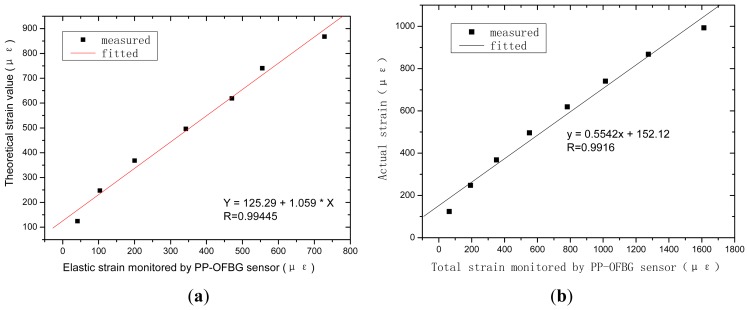
Strain of asphalt beams in static load test: (**a**) Theoretical strain; (**b**) Actual strain.

Measured and actual strains are drawn in Figure 17. From the results we can say that measured strain matches linearly with the actual one. The correlation coefficient was 0.9916, although maybe due to the position of the sensors in the specimens, the two fitted equations show a small difference.

## 6. Conclusions

Aiming at measuring and monitoring low modulus materials or structures such as asphalt concrete pavement, a novel polypropylene based OFBG strain sensor was designed and produced. Its micro-structure, mechanical, strain sensing properties were also studied. The strain sensing coefficient is about 0.85 pm/με, which is lower than that of bare OFBG. This is mainly because of the micro-mechanics interface strain delivery. The results show that the interface of PP matrix and bare OFBG is very good and this new PP-based OFBG sensor has a good sensitivity with low modulus, which can meet the requirements of the strain measurement or monitoring of low modulus materials or structures such as asphalt pavement.

## Figures and Tables

**Table 1.** Properties of polypropylene.

**Appearance**	**Melt Flow Index** **(g/10 min)**	**Isotactic Index** **(%)**	**Apparent Density** **(g/cm^3^)**	**Tensile Yield Strength** **(MPa)**	**Processing Temperature** **(°C)**
white	37∼50	96	0.41	31.5	180∼240

**Table 2.** Properties of maleic anhydride drafted polypropylene.

**Brand**	**Appearance**	**Melt Flow Index(g/10 min)**	**MAH Graft Content (%)**
BonpTM-GPM200A	white	160∼180	1.4

**Table 3.** Temperature control of PP based OFBG strain sensor fabricating process.

	**Zone 1**	**Zone 2**	**Zone 3**	**Zone 4**
**Temperature (°C)**	180	200	220	100
